# Extravesical Ureteral Reimplantation Following Lich-Gregoir Technique for the Correction of Vesico-Ureteral Reflux Retrospective Comparative Study Open vs. Laparoscopy

**DOI:** 10.3389/fped.2018.00388

**Published:** 2018-12-18

**Authors:** Nasir Bustangi, Anthony Kallas Chemaly, Aurelien Scalabre, Karim Khelif, Stéphane Luyckx, Henri Steyaert, Francois Varlet, Manuel Lopez

**Affiliations:** ^1^Department of Pediatric Surgery and Urology, King Abdulaziz University Hospital, Jeddah, Saudi Arabia; ^2^Department of Pediatric Surgery and Urology, Faculty of Medicine, Hôtel-Dieu de France, Beirut, Lebanon; ^3^Department of Pediatric Surgery and Urology, Faculty of medicine, Hôtel-Dieu de France Hospital, Université Saint-Joseph, Beirut, Lebanon; ^4^Queen Fabiola Children's University Hospital, Bruxelles, Belgium; ^5^Department of Pediatric Surgery and Urology, Hospital Universitari Vall d'Hebron, Barcelona, Spain; ^6^University Hospital of Saint Etienne, France

**Keywords:** vesicoureteral reflux, comparative study, open, laparoscopy, Lich Gregoir

## Abstract

**Introduction:** The aim is to compare the outcome of open versus laparoscopic Lich-Gregoir technique in patients with vesicoureteral reflux. We report a retrospective multicenter comparative study between open and laparoscopic extra-vesical ureteral reimplantation (EVUR) following Lich-Gregoir (LG) technique for the correction of Vesico-Ureteral Reflux (VUR).

**Materials and Methods:** Between January 2007 and December 2015, 96 patients with VUR (69 females and 27 males) and deterioration of the renal function, underwent EVUR following LG technique. Fifty patients (16 males and 34 females) were operated by open surgery (group A). The mean age was 4.22 years-old, (14–147 months). Laparoscopic approach (group B) was performed in 46 patients (11 males and 35 females). The mean age was 4.19 years-old (15–110 months). We compared the results in relation to degree of VUR, operative time, hospital stay, post-operative pain medications, recovery time, complications, successful rate, recurrence, and follow-up. Statistical analysis was done used Chi square test for categorical variables and the Student *t*-test for continuous variables. *P* < 0.05 was considered significant.

**Results:** In both groups no correlation was identified between age or weight and operative time, length of stay or total analgesia used. The mean operative time for group A was 63.2 and 125.4 min for unilateral and bilateral VUR, respectively, and for the group B was 127.90 and 184.5 min, respectively. There was no conversion in the laparoscopic group. Perioperative mucosal perforation of the bladder occurred in 6 patients of group A and 4 patients of group B and was immediately repaired. One patient had to be reoperated for leakage in group B. The mean duration of Morphine, IV and PO analgesia was shorter in group B. The mean hospital stay was 5.46 days for group A and 1.54 days for Group B. The success rate was 98% in group A and 97, 8% in group B. The mean follow-up was 3.67 years for the open and 1.54 years for the laparoscopic group. Transitory voiding dysfunction occurred in bilateral EVUR in one case in each group.

**Conclusion:** Laparoscopic or Open approach for the correction of VUR following Lich-Gregoir technique is effective in unilateral and bilateral VUR with similar results. Laparoscopic approach reduces significantly (*p* < 0.05 in each item) post-operative pain medication, hospital stay, and allows for a faster return to normal activity.

## Introduction

Vesicoureteral reflux (VUR) represents one of the most significant risk factors of acute pyelonephritis (APN) in children. Nephropathy with renal scarring is still the most concerning issue in VUR (1). Early detection and monitoring of VUR are the cornerstones of management and kidney protection. Evaluation of VUR treatment outcomes should consider not only resolution of VUR over time but also disappearance of urinary tract infections (UTI) and evolution of renal scars.

Several options exist nowadays for the treatment of VUR including: Surveillance program with or without antibiotic prophylaxis, endoscopic, laparoscopic, and open approach. Surgical correction to eliminate VUR is an important part of the management (2). Intravesical or Extra vesical technique have been described for the correction of VUR with a high success rate (3). Regarding intravesical approach, Ledbetter-Politano and the Cohen technique have been considered the most popular techniques of ureteral reimplantation with successful rate in the range of 97–99% (3).

EVUR was described by Willy Gregoir in 1961 and 1964 (4). It is an excellent technique with similar successful rates to the intravesical approach especially when it's combined with some modifications described in the literature (5–7). Notwithstanding this operation gained poor acceptance in Europe due to the risk of denervation of the bladder, at least in bilateral cases (8).

Minimally invasive surgery (MIS) for the correction of VUR is being developed as an alternative to open surgery. Nevertheless, the real concept of MIS in this field was introduced by O'Donnell and Puri with the endoscopic sub-ureteric injection procedure (9, 10). Most studies would nevertheless suggest this approach has not become the standard because it has a lower success rate particularly in high degree of VUR (11, 12). Pneumovesicoscopic MIS have been a challenge even for the more experienced surgeons. Cohen MIS has never been achieved popular because of the technical difficulty in dissection and suturing (13).

The introduction of EVUR by MIS was described by Atala et al. in minipigs (14), but the first report in humans was described in 1994 by Ehrlich et al. (15).

After that different reports have been published reporting a successful rate similar to open procedures (16). At our knowledge, we report the first comparative study between open and laparoscopic EVUR following the LG technique for the correction of VUR in two European centers.

## Materials and Methods

### Patients Selection and Study Design

A retrospective study was conducted in two referral centers in Europe (France and Belgium), studying charts of patients operated using the LG technique from January 2007 to December 2015. Patient's data were obtained from the medical records after institutional board approval in each center.

Exclusion criteria were patients with primary obstructed mega-ureter or refluxing mega-ureter needing a tapering. Charts evaluation included: Prenatal diagnosis, history of UTI, antibiotic prophylaxis, ultrasound (US), Voiding cystourethrogram (VCUG), renal Scintigraphy (RS), symptoms of bladder dysfunction, and previous endoscopic treatment.

During perioperative evaluation we compared: type of anesthesia, associated procedures, perioperative complications, conversions, and operative time.

In the postoperative period the evaluation included: complications, urinary retention, time of Morphine/Nalbuphine, duration of oral analgesia, and hospital stay.

UTI were also searched for and divided as: early UTI (within the first month) and late UTI (more than 1 month after surgery).

Follow-up was recorded in all patients. In between 6 weeks post-operatively, renal and bladder US was retrieved for all patients.

In the laparoscopic group, for the first 30 cases, a VCUG, was systematically performed in order to validate the efficacy of the surgical technique. Actually in both group, a VCUG is only indicated in case of recurrent UTI.

The successful rate was defined by the absence of documented febrile UTI or absence of recurrence of VUR objectivized by VCUG in both groups. RS was done depending on team preferences and not systematic but all cases had decreased renal function preoperatively. Statistical analysis was done used Chi square test for categorical variables and the Student *t*-test for continuous variables. A *p*-value of < 0.05 was considered statistically significant.

### Technique Description

**Figure 1:**
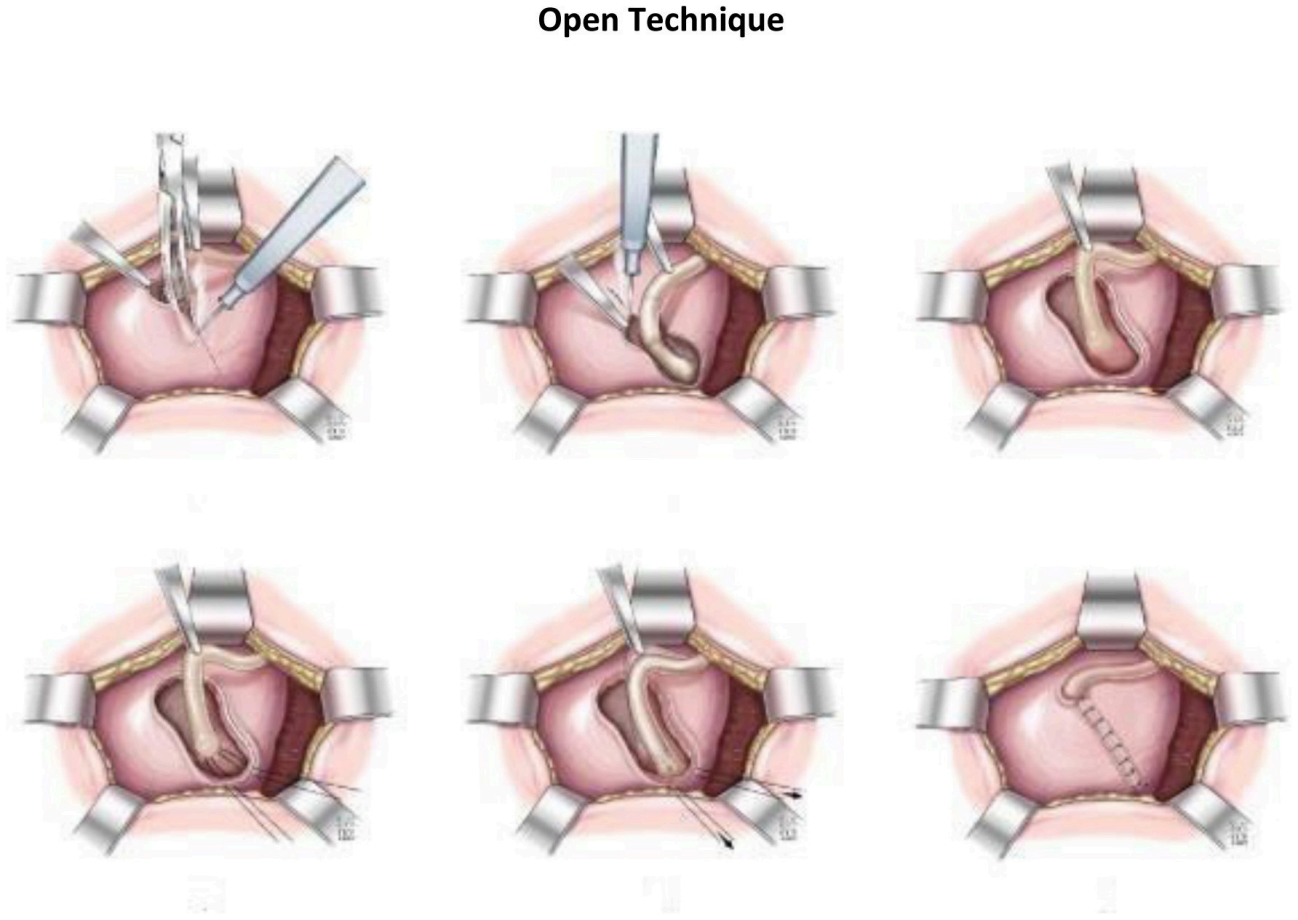
Open technique.

In open surgery, a half of complete pfannenstiel incision was done, with an extraperitoneal approach to the bladder. Y-shaped incision proximally to release the flaps longitudinally and allow complete embedding. The ureter is placed in the new tunnel and reapproximated with interrupted reabsorbable 3.0 suture (Figure 1).

**Figure 2:**
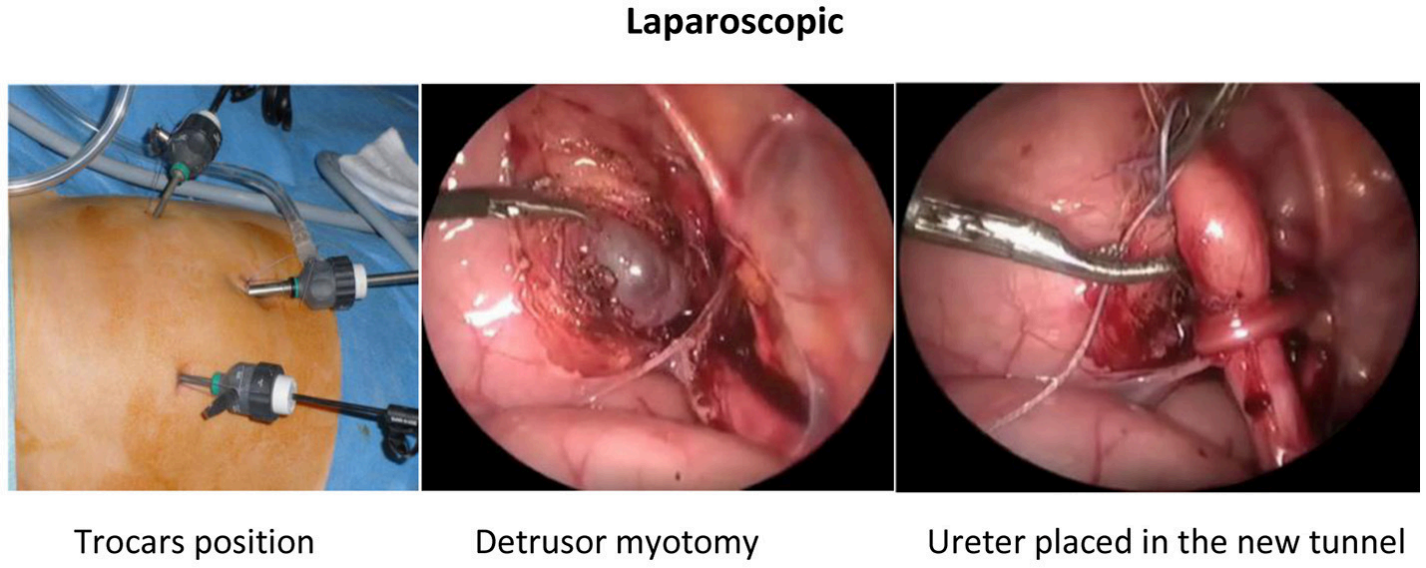
Laproscopic.

In laparoscopic approach, the patient was placed in supine position with the legs apart. Three ports were used in all cases, 5 mm 30° for the telescope and two 3-mm trocars. The surgeon was positioned at the head of the patient, with the assistant to the left and the nurse to the right. After trocar positioning and pneumoperitoneum, stay sutures were used to expose the vesicoureteral junction (VUJ). Two stay sutures were inserted through the abdominal wall and placed in each side of the bladder to pull it up to the anterior wall and expose the VUJ. The peritoneum was incised just to identify the distal ureter that was isolated and dissected toward the VUJ. The ureter was mobilized to achieve sufficient length for a tension-free reimplantation. A vertical Detrusor myotomy was performed by using the monopolar scissors to create an optimal lateral tunnel with a length about 4 times the size of the ureter (Paquin law). At this moment, the bladder was filled partially with physiologic serum. The detrusor muscle and all muscle fibers were cautiously divided down with scissors until the mucosa was exposed. After completing the dissection, another stay suture was inserted through the abdominal wall and placed around the ureter toward the top of the bladder. The ureter was placed in the newly created tunnel, and the detrusor muscle was reapproximated with 3 or 4 separate intracorporeal stitches with absorbable sutures 3-0 (Figure 2).

## Results

From January 2007 to December 2015 a total of 119 renal units in 96 patients (69 females and 27 males), with VUR with recurrent UTIs and deterioration of renal function at RS, underwent EVUR following the LG technique. Operation was done after failure of endoscopic treatment in 4 and in 3 patients in group A and group B, respectively.

Open surgery (group A) was performed in 50 patients (16 males and 34 females). The mean age at operation was 4.2 years-old (14–147 months). Laparoscopic EVUR (group B) was done in 46 patients (11 males and 35 females). The mean age was 4.2 years-old (15–110 months) (Table 1).

**Table 1:** Patient and surgical demographics.

**Patients**	**Open**	**Laparoscopy**	*P-*value^*^
Numbers	50	46	
Sex		
Male, *n*(%)	16 (32.0)	11(23.9)	0.4
Female, *n*(%)	34 (68.0)	35 (76.1)
Mean age, years (SD)	4.22 (2.78)	4.19 (2.61)	0.6
Mean weight, kg (SD)	12.24 (12.38)	16.20 (5.90)	0.06
Prenatal UTD, *n* (%)	21 (42.0)	10 (21.7)	0.03
Unilateral RVU, *n* (%)	38 (76.0)	35 (76.1)	0.9
Right, *n* (%)	13 (35.1)	8(22.9)	0.3
Left, *n* (%)	24 (64.9)	27(77.1)	0.7
Grade III	25 (65.7)	23 (65.7)	0.7
Grade IV	13 (34.3)	12 (34.3)
Bilateral RVU, *n* (%)	12(24.0)	11(23.9)	1
Grade II, number of kidneys (%)	3 (12.5)	1 (4.5)	0.35
Grade III, number of kidneys (%)	11 (45.8)	13 (59.1)	0.35
Grade IV, number of kidneys (%)	8 (33.3)	6(27.3)	0.35
Grade V, number of kidneys (%)	2 (8.3)	2 (9.1)	0.35

* *P-value calculated using the Chi square test for categorical variables and the Student t-test for continuous variables. UTD, urinary tract dilatation; SD, standard deviation*.

Prenatal diagnosis was found more frequently in Group A (21 vs. 10) (*P* < 0.03).

In both groups, preoperatively all patients were under antibioprophylaxis. All suffered from 2 or more pyelonephritis. There was no difference between the groups in terms of preoperative voiding dysfunction.

In the perioperative period, 94% in group A had epidural anesthesia and 6% caudal anesthesia. In group B, 39% had a transversus abdominis plane block.

Cystoscopy was associated more often in group B (22 vs. 5), (*p* < 0.0001).

During detrusor myotomy, mucosal perforation occurred in 6 and 4 patients in group A and B, respectively. The mean operative time was significantly longer in the laparoscopic group with 127.90 and 184.5 min for unilateral and bilateral EVUR, respectively while in group A it was 63.2 and 125.4 min for unilateral and bilateral cases, respectively (Table 2).

**Table 2:** Perioperative and postoperative outcomes.

	**Group A**	**Group B**	*P*-value^*^
Mean operative time, min (SD) For Unilateral	63.2 (12.70)	127.90 (36.83)	< 0.0001
Mean operative time, min (SD) For Bilateral	125.4 (26.36)	184.5 (46.1)	0.007
Perioperative mucosal perforation, *n* (%)	6 (12.0)	4 (8.7)	0.59
Post-operative urinary retention, *n* (%)	1 (2)	1 (2.1)	0.34
Early febrile UTI (<30 days after surgery), *n* (%)	0 (0)	3 (6.5)	0.07
Late Febrile UTI, *n* (%)	5 (10.0)	3 (6.5)	0.5
Mean morphine or nalbuphine treatment duration, days (SD)	3.24 (0.80)	0.52 (0.50)	< 0.0001
Mean intravenous analgesia duration, days (SD)	3.98 (1.09)	1.15 (0.50)	< 0.0001
Mean PO analgesia duration, days (SD)	9.58 (3.68)	2.48 (1.69)	< 0.0001
Mean hospital stay duration, days (SD)	5.46 (1.54)	1.64 (1.03)	< 0.0001
Mean follow-up, years (SD)	3.67 (1.78)	1.54 (1.30)	< 0.0001

* *P-value calculated using the Chi square test for categorical variables and the Student t-test for continuous variables. SD, standard deviation*.

Surgical procedures were performed without conversion in the laparoscopic group.

During the postoperative period the mean duration was significantly shorter in group B for Morphine/Nalbuphine (3.24 vs. 0.52 days) (*p* < 0.0001) and for oral analgesia (9.58 vs. 2.48 days) (*p* < 0.0001).

One patient in group B presented ureteral leakage at day 8 postop needing a redo-laparoscopic procedure after readmission. The mean hospital stay was significantly shorter in the group B (1.64 days vs. 5.46).

Transitory voiding dysfunction occurred in bilateral EVUR in one case in each group. Resolution required bladder reeducation for 5 weeks in the group A case and resolved spontaneously after 1 week in the group B case. There was never a need for urinary catheter drainage.

Early febrile UTI occurred in 2 cases in the group B, and were treated by intravenous antibiotic with uneventful course. Late febrile UTI occurred in five patients in group A and in three patients in group B. In these cases a VCUG was done and confirmed the persistence of VUR grade III in only one patient in group A, who needed a redo procedure following the Paquin-Mollard technique.

The overall follow-up period was significantly longer in the open group (3.67 vs. 1.54 years) (Table 2). The success rate was 98 and 97.8% in the group A and B, respectively.

## Discussion

In the past 30 years, the therapeutic approach for children with VUR has undergone a dramatic evolution. Surgery in primary intention toward a conservative approach with active surveillance, with or without antibiotic prophylaxis. Afterward minimally invasive approach using endoscopic or laparoscopic procedure (17–22).

The management of VUR continues to evolve. Endoscopic treatment of VUR is still considered the first line of treatment in many centers even for high grade reflux, although treatment has frequently to be repeated in order to obtain the same percentage of success as reimplantation techniques (23).

Analyzing the literature, it becomes more and more evident that the decision for treating VUR and the type of treatment in a child is an individualized process (17).

In open surgery, the LG technique seems to be associated with less discomfort and allows earlier mobilization than intravesical surgery (24). Different techniques using MIS to treat VUR demonstrated their feasibility and efficacy: pneumovesicoscopic, laparoscopic, or robotic-assisted (13). They are encouraging and have been reported to be beneficial in terms of decreased postoperative pain, shorter hospital stay, and quicker return to normal activity (16).

A recent publication compared endoscopic, laparoscopic and open surgery for the treatment of VUR. Open Cohen and laparoscopic treatment using LG technique had higher success rates than STING procedure. However, Cohen had a longer hospital stay, more complications and analgesic requirements compared to STING and Laparoscopic EVUR (25). LG technique by laparoscopy was described using a trans-peritoneal approach. Recently tips and tricks of this technique has been reported, in order to decrease the perioperative morbidity and to be reproducible for young surgeons in training (26).

The reoperation we had in group B explains also partly the length of stay in this group. Excluding this case, the mean hospital stay for a laparoscopic LG (without complications) is about 1.64 days. In 2008 and 2009, Palmer demonstrated that unilateral and bilateral EUVR could be performed on a day-surgery basis (27, 28). Longer hospital stay in the open group in this series is more related to habits and reimbursement system than to a real necessity. Recently system changed and hospital stay shortened dramatically.

Main concern in the LG technique is the risk of voiding dysfunction. Several series reported a bladder voiding dysfunction with an incidence ranging from 3 to 20% in different series (29, 30). It might be the result of neurovascular injury during ureteral or bladder dissection (31). However, more recently, McAchran and Palmer demonstrated that in bilateral cases, surgical correction can be performed without postoperative urinary retention (32). Yucel and Baskin described the neuroanatomy of the distal ureter, UVJ and their clinical application. They showed that nerves occupy the medial aspect of the distal ureter and that, at level of UVJ, the nerves encircle the entire ureter. They travel just outside Waldeyer's sheath, leaving a safe area for surgical dissection under this sheath (33). Our series demonstrates, too, that voiding dysfunction is rare certainly in unilateral cases. We encountered very few problems in the two groups (1 case in each).

Peters et al. showed a comparative outcome between open Cohen and robotic assisted EVUR with longer operative time in the robotic group (34).

In the robotic approach, Peters in 2004 reported a voiding dysfunction in one case (5, 8%) (34). Randomized trials could be necessary to see if a MIS or robotic approach could help to decrease the risk of bladder dysfunction but numbers of patients operated for VUR is becoming so small that this task is probably impossible to accomplish. Considering that authors conducted a retrospective multi-center (two) study, follow up and treatment options before the Lich Gregoir were different and institution dependent. This is part of the limitations due to this type of studies. In duplicated collecting systems, the LG sheath has shown an excellent results in the literature (35). In our study we had more than 30 cases with duplicated collecting systems with excellent results in both groups.

## Conclusion

We demonstrate that MIS LG is effective in unilateral and bilateral VUR with a similar success rate as in open surgery. Laparoscopic approach reduces post-operative pain medication and permits a faster return to normal activity. Day surgery is to be considered as a perfectly attainable objective in the MIS as well as, probably, in the open approach. The neuroanatomy of bladder, ureters and VUJ should be kept in mind for this technique.

## Author Contributions

NB: writing the manuscript and references; NB and AK: responsable about data collections and framing; AS: did the statistical analysis; HS, FV, and ML: study design and ethical approval. All authors included in the final revision and approval of the finalization.

### Conflict of Interest Statement

The authors declare that the research was conducted in the absence of any commercial or financial relationships that could be construed as a potential conflict of interest.
